# Influence of mental stress on the pulse wave features of photoplethysmograms

**DOI:** 10.1049/htl.2019.0001

**Published:** 2019-11-26

**Authors:** Patrick Celka, Peter H. Charlton, Bushra Farukh, Philip Chowienczyk, Jordi Alastruey

**Affiliations:** 1 Polar Electro Oy, Professorintie 5, 90440 Kempele, Finland; 2 Department of Biomedical Engineering, School of Biomedical Engineering and Imaging Sciences, King's College London, King's Health Partners, London SE1 7EH, UK; 3 King's College London British Heart Foundation Centre, Department of Clinical Pharmacology, King's College London, King's Health Partners, St. Thomas’ Hospital, London SE1 7EH, UK; 4 Institute of Personalized Medicine, Sechenov University, Moscow, Russia

**Keywords:** photoplethysmography, cardiology, medical signal processing, medical disorders, pulse wave features, arm photoplethysmogram, wave shape parameters, stress assessment, mental stress, pulse transit time, PPG, dicrotic notch, end diastole, heart rate variability

## Abstract

Mental stress is a major burden for our society. Invasive and non-invasive methods have been proposed to monitor and quantify it using various sensors on and off body. In this Letter, the authors investigated the use of the arm photoplethysmogram (PPG) to assess mental stress in laboratory conditions. Results were in correspondence with their previous *in-silico* study which guided the present study. Three wave shape parameters were identified for stress assessment from the PPG signal: (i) the time from dicrotic notch to end diastole; (ii) the time from pulse onset to systolic peak; and (iii) the ratio of diastolic to systolic area. The proposed in-vivo results showed that the two first parameters responded significantly to increased mental stress and to a breathing relaxation procedure, complementing heart rate, heart rate variability, and pulse transit time as indices of stress.

## 1 Introduction

Mental stress is a harmful psychological reaction to an increased level of demand placed on a person. It is a common health issue, associated with increased cardiovascular mortality and morbidity [1]. It also has a great impact on both professional and private lives. As an example, stress (we will use the term stress in place of mental stress in this Letter) in the workplace costs British industry billions of pounds per year, and together with depression and anxiety, accounted for 12.5 million lost working days per annum in 2017 [2]. Stress also affects individuals, being associated with negative mood [3], immunosuppression [4], impacts on physical and mental health and increased occurrence of illnesses [5]. Several stress management interventions have been shown to be effective in both the workplace and personal settings [6]. This provides great incentive for developing techniques to recognise elevated stress levels, prompting interventions to reduce stress levels, and potentially improve health.

Several techniques are currently used to assess stress levels, although these are limited to intermittent measurements often requiring a skilled operator, rather than being suitable for continuous monitoring. For instance, cortisol hormone levels, which increase during stress, can be measured from samples of saliva, blood or urine [7]. Stress can also be assessed from cardiovascular parameters such as blood pressure (BP), heart rate (HR), and heart rate variability [8]; respiratory parameters [9]; galvanic skin response [10]; and biofeedback systems used to train athletes [11]. However, changes in these parameters are not specific to stress. Therefore, there is a need to develop techniques for continuously monitoring stress levels.

It has recently been proposed that stress could be assessed from the photoplethysmogram (PPG), a non-invasive signal which captures changes in blood volume over time in a bed of tissue [12–15]. PPG signals have been used in clinical assessment for several decades and can now be measured by consumer products such as smart watches and sports equipment. The PPG signal contains information about heart muscle function, the circulatory system, blood flow and its constituents, blood perfusion (which is linked to body temperature), BP and its variability, the autonomic nervous system (ANS, both central and local, through the variability of its amplitude and the inter-beat intervals), and respiratory function [9]. The effect of relaxation response on endothelial function has also been shown, linking stress to vascular function [16]. In addition, the PPG can be recorded at different wavelengths to give information about superficial and deep tissue structures, and micro- and macro-circulation simultaneously [17]. Consequently, several aspects of the PPG signal may change with stress, providing opportunity to develop a robust PPG-based stress index by fusing several cardiovascular parameters extracted from the PPG signal. Such an index could be used to monitor stress levels continuously, using everyday consumer devices.

We have previously reported a numerical study in which three PPG pulse wave features were found to change with haemodynamic changes associated with stress, and were identified as candidate features for in-vivo testing [18]. The model simulated pulse wave propagation in the larger arteries of the systemic circulation, including the larger arteries of the arms and head. In the brachial, radial, and temporal arteries, BP signals were simulated and transformed into PPG pulse waves using a transfer function. PPG pulse waves were simulated at six levels of stress by adjusting the following model input parameters to mimic haemodynamic changes which occur with stress: HR, stroke volume, left ventricular ejection time, systemic vascular resistance, and arterial stiffness. The following three pulse wave features showed significant trends at the brachial, radial, and temporal sites: the time from pulse wave onset to crest (CT), the duration of the diastole ($t_{{\rm dia}}$), and the ratio of the area under the pulse wave curve during diastole and systole (IPA).

This Letter presents an in-vivo study of changes in PPG pulse wave features in response to stress in healthy subjects during a controlled laboratory stress and relaxation protocol. The aim was to determine whether the three previously identified features (CT, $t_{{\rm dia}}$, and IPA) change with stress as predicted by the *in-silico* study. These features were compared with commonly used parameters to quantify cardiovascular stress: HR, pulse transit time (PTT), and the root mean square of the successive difference of inter-beat intervals (RMSSD), a heart rate variability index correlated with parasympathetic activity [19, 20]. Throughout the Letter, we have used features’ names without a hat to refer to either the *in-silico* values or the concepts in general, while the features with a hat refer to statistical estimates from our in-vivo experiments.

## 2 Materials and methods

### 2.1 Subjects

Ten young, healthy subjects (four males and six females, age range 23–31 years, BMI range 17.6–33.8 kg m^−2^) participated in the study at St. Thomas’ Hospital, London. All subjects completed a preliminary questionnaire about cardiovascular and mental health as well as any medications that could influence the results. Exclusion criteria were: diagnosed hypertension, heart arrhythmias, cognitive impairments. The *NRES Committee London – Westminster* approved the study. Subjects could ask to withdraw or pause at any time during the study.

### 2.2 Protocol

**Fig. 1:**
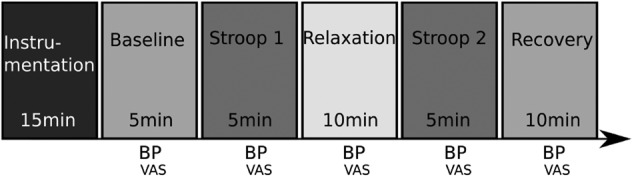
Six phases of the stress study. BP: cuff blood pressure measurement; VAS: visual analogue scale for subjective stress assessment

The study protocol consisted of six phases as illustrated in Fig. 1: instrumentation, baseline measurements, Stroop test 1, relaxation phase, Stroop test 2, and recovery. BP measurements and subjective stress assessment using a visual analogue scale (VAS) were performed before and after each protocol phase. The study was conducted in a dedicated room, isolated from noise and other visual disturbances. The study phases are described next.

During the first, *instrumentation phase*, subjects were provided with instructions on how to perform the Stroop test and relaxation phases, and measurement instruments were attached (as explained in the next section). As part of the routine clinical protocol at the hospital, subjects completed the Patient Health Questionnaire (PHQ-9) [21]. The second, *baseline phase*, consisted of acquiring measurements from subjects whilst lying on a bed, head tilted up slightly, for 5 min whilst breathing spontaneously. In the third, *Stroop test 1 phase*, stress was induced using the colour word Stroop test [22]. This test has been shown to provide reasonable results in terms of controlled induced stress and is widely used in psychology research. The test was performed for 5 min while subjects were lying down in the bed looking at a computer screen where the Stroop test was running. Subjects were asked to answer simple word-colour-matching questions, at an increasingly faster pace as the test progressed to compensate for the known adaptation process that subjects undergo. In the fourth, *relaxation phase*, subjects used the Resperate system (Resperate, Inc) for 10 min, which is designed to lower BP through device-guided slow-paced breathing [23]. The breathing frequency range was adjusted for each individual according to their comfort zone. In the fifth, *Stroop test 2 phase*, a second Stroop test was conducted lasting 5 min. In the sixth, *recovery phase*, subjects relaxed, unaided and in silence, for 10 min whilst isolated by a curtain.

Reference assessments of stress were obtained at the end of each phase by asking subjects two questions: (i) do you feel any pain or discomfort?; and (ii) how would you rate your stress level? Subjects provided responses using a VAS ranging from 0 to 10. The VAS has been successfully used in many psychological studies and has the great advantage of being very simple, especially during experiments when subjects are psychologically stressed [24].

### 2.3 Signal acquisition

The SOMNOtouch RESP system (SOMNOmedics, GmbH) was used during the entire protocol to acquire cardio-respiratory measurements. It recorded 3-lead electrocardiogram (ECG, 2 leads below clavicles and 1 lead at the waist level, sampling frequency 1 kHz), and arterial blood oxygen saturation from a finger sensor together with a PPG signal (Nonin sensor, at 128 Hz). The beat-by-beat PTT was extracted from the ECG and finger PPG signals in a post-processing phase by the SOMNOmedics NIBP software. The SOMNOmedics software also provided inter-beat-intervals derived from the ECG (IBI-ECG). The SunTech Oscar 2 BP instrument (SunTech Medical, Inc) was used to measure BP from the left arm.

**Fig. 2:**
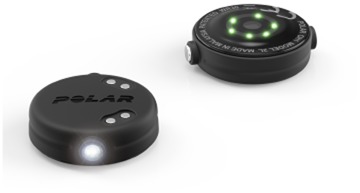
OH1 photoplethysmograph (PPG) sensor. (top image) The hexagonal structure with the six green LEDs and the central photodiode, together with the button for initiating a recording. (bottom image) The four connectors for battery charging, data transfer, together with a colour LED for device status

PPG signals for pulse wave analysis were acquired using OH1 sensors (Polar Electro Oy). The OH1 device complies with electro-magnetic radiation safety, has been tested for skin biocompatibility, and was CE marked. The OH1 sensor consists of a hexagonal arrangement of green light sources and measures multiple PPG signals at 135 Hz. Six PPG signals were acquired simultaneously by the sensor and an averaging technique was used to increase the signal to noise ratio. Fig. 2 shows the OH1 PPG sensor (25 mm diameter × 7 mm height).

Three OH1 sensors were used to acquire PPG signals simultaneously at different locations: the left wrist (dorsal side), the left upper arm (lateral side), and the left temporal region. Inter-beat-intervals derived from the PPG (IBI-PPG) were extracted by a proprietary algorithm (Polar Electro Oy) [25]. The IBI-PPG was used for the pulse wave parameter extraction and statistical analysis.

### 2.4 PPG signal processing

**Fig. 3:**
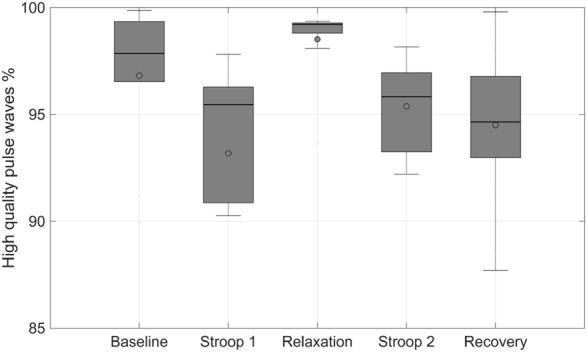
Statistical distribution of the percentage of high-quality PPG pulse waves for each subject and each protocol phase. Box plots show median and lower and upper quartiles

PPG signals are very sensitive to movement artefact and other sources of noise. Therefore, for each subject, and for each protocol phase, the quality of the PPG signal was assessed visually by an expert and high-quality segments were selected for further analysis. Segments were deemed to be of high quality if the dicrotic notch could be identified from the original signal guided by the shape of its first and second derivatives. The proportion of pulse waves which were of high quality for each subject at each protocol phase is illustrated in Fig. 3. On average, over 90% of pulse waves were of high quality in each phase. The highest percentage was observed in the relaxation phase, followed by the baseline phase. The percentage of high-quality pulse waves was lower in the Stroop and recovery phases. The small inter-quartile range in the relaxation phase percentage indicates that this phase had the effect of stabilising the PPG signal.

**Fig. 4:**
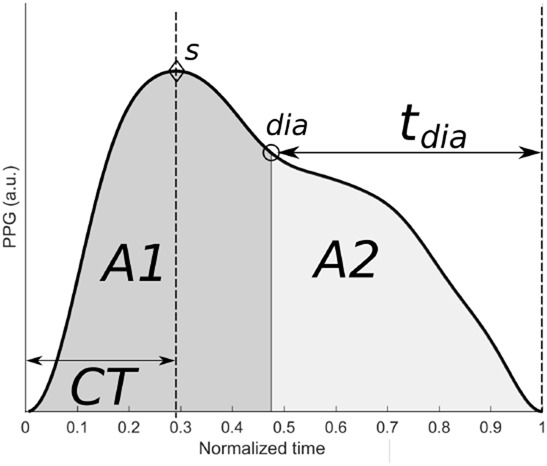
Averaged pulse wave, $\overline {PW} _n\lpar t\rpar $, from ten consecutive pulse waves acquired at baseline together with the fiducial points *s* and dia, and features CT, $t_{dia}$, $A1$ and $A2$, which are described in the main text

An averaged pulse wave was extracted as follows. Firstly, pulse wave onsets (at the beginning of systole) were identified using the first and second derivatives of the PPG in the vicinity of the detected pulses used for the estimation of the IBI-PPG. A sequence of $N = 40$ pulse waves, $PW_k\lpar t\rpar $, $k = 1...\comma \; N$, was selected. This allowed us to compute an average waveform, as described below, for each phase of the protocol. Due to the non-stationarity of the PPG signal, we tried to select a series of pulse wave $PW_k\lpar t\rpar $ which covered each entire protocol phase as uniformly as possible. These 40 waves were then accumulated in a vector. Breathing also affects waveform shape [26], and this effect was hopefully reduced by the averaging procedure. The averaging procedure was carried out as follows. Each pulse wave was time-scaled on a uniformly resampled time axis covering 1 s at a sampling frequency of 750 Hz using spline interpolant to increase the precision of fiducial-point identification. An averaged PPG pulse wave was computed from each set of $L = 10$ consecutive pulse waves, and repeated $N - L + 1$ times by incrementing the pulse wave number *k* by one resulting in a series of $N - L + 1 = 31$ averaged pulse waves, $\overline {PW} _n\lpar t\rpar $, $n = 1\comma \; ...\comma \; N - L + 1$. Fig. 4 shows an example of an averaged pulse wave, $\overline {PW} _{n = 1}\lpar t\rpar $.

Pulse wave features were extracted from each averaged pulse wave, $\overline {PW} _n\lpar t\rpar $, as follows. Two fiducial points were identified on each $\overline {PW} _n\lpar t\rpar $: the systolic peak, *s*, and the dicrotic notch, *dia* (see Fig. 4), using the waveform maximum and its second derivative minimum of $\overline {PW} _n\lpar t\rpar $, respectively. During the time-scaling normalisation procedure, each scaling coefficient was stored and the average was used to estimate the true position of the time location of *s* and *dia* for each $\overline {PW} _n\lpar t\rpar $. Three pulse wave features were then extracted for each $\overline {PW} _n\lpar t\rpar $: the crest time (CT, i.e. time from pulse onset to systolic peak); the duration of diastole ($t_{{\rm dia}}$, i.e. time from dicrotic notch to pulse end): and the inflection point area ratio [${\rm IPA} = A2/A1$, i.e. ratio of diastolic area (light grey), $A2$, to systolic area (dark grey), $A1$]. Fig. 4 shows the identified fiducial points and pulse wave features.

### 2.5 Comparison with previous *in*-*silico* results

In our previous numerical study [18], we observed that CT, $t_{{\rm dia}}$, and IPA changed with stress when PPG pulse waves were simulated using the one-dimensional (1D) formulation of pulse wave propagation [27]. Six different stress levels were simulated: *baseline* (simulated stress level 0, *SSL-*0, representing baseline state), *relaxation* (*SSL*-1, reduced stress), and four levels of increasing stress (*SSL-*1–*SSL-*4). Different stress levels were simulated using different values for HR, cardiac output and systemic vascular resistance (see Table 2 in [18]), resulting in different BP values. Protocol phase stress levels in the present study were matched to the SSLs from the previous study using the measured values of HR and BP to produce the corresponding stress levels shown in Table 1. We arbitrarily imposed the *recovery* phase to be the average of *SSL-*0 and *SSL-*4 to take into account the adaptation process that subjects undergo after a stress test.

**Table 1:** In-vivo and in-silico stress levels correspondence

Protocol phase (in vivo)	Simulated stress level (in silico)
relaxation	SSL-1
baseline	SSL-0
Stroop 1	SSL-2
Stroop 2	SSL-4
recovery	$\lpar \hbox{SSL-0 + SSL-4\rpar /2}$

### 2.6 Statistical analysis

Distributions of parameters across subjects were summarised using the median and inter-quartile range in box plots. Mean values were also reported. Significant differences between parameters measured in the baseline and other protocol phases were identified using paired *t*-tests (significance level $\alpha = 0.05$). Estimated pulse wave parameters are denoted with hats to distinguish them from their theoretical values obtained from the *in-silico* study.

## 3 Results

### 3.1 Pulse wave shape

The PPG signal quality at the wrist and temporal region was too low on average to extract robust statistics and draw meaningful conclusions. The wrist (dorsal side) is known to be one of the less reliable places for measuring good quality PPG signals whilst still providing useful HR information in many commercial wearables. The temporal artery was easier to locate but the PPG sensor was occasionally displaced during the Stroop tests as the subject had to move their head to look at a computer screen. We thus present only results from PPG signals acquired at the left arm, which were of consistent high quality across subjects and protocol phases.

**Fig. 5:**
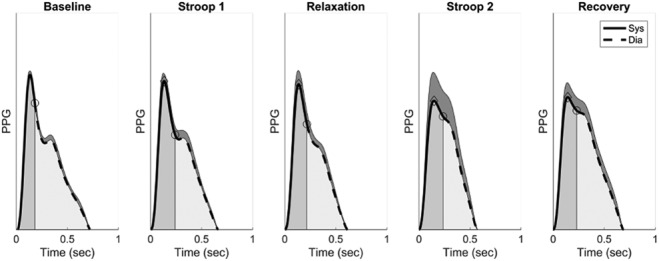
Example of arm PPG pulse wave shapes from baseline to recovery. The average pulse wave, $\overline {PW} _{n = 1}\lpar t\rpar $, and standard deviation (shaded areas on top of the average waves) are displayed together with systolic (dark shaded areas) and diastolic (light shaded areas) phases. Points indicating systolic peaks (diamonds) and the start of diastole (circles) are also shown

**Fig. 6:**
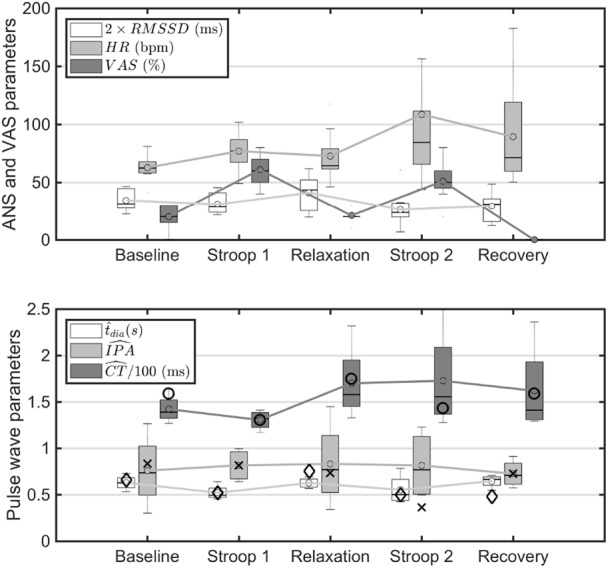
Influences of stress on ANS, VAS, and pulse wave shape parameters. (top) Changes in ANS parameters. HR: heart rate; RMSSD: root mean square of the successive difference of inter-beat intervals (scaled by a factor of two), and VAS. (bottom) Changes in estimated pulse wave features ($\hat t_{dia}$: time from dicrotic notch to end diastole; $\widehat{{IPA}}$: ratio of diastolic to systolic area; $\widehat{{CT}}$: time from pulse onset to peak). Corresponding scaled pulse wave parameters from our numerical simulations [18] are shown with the following symbols: diamonds ($\diamond $) correspond to $1.372\ast t_{dia}$, crosses ($ \times $) to $0.907\ast IPA$ and circles ($ \circ $) to $0.795\ast CT$

The arm PPG signal showed recognisable pulse wave features, as illustrated in Fig. 5. The pulse wave shape changed with stress, as observed in our previous study [18]. Two observations were drawn from Figs. 5 and 6. Firstly, the HR increased during the Stroop tests, impacting directly on $t_{{\rm dia}}$ and CT. Secondly, breathing affected the pulse wave shape: (i) the averaging on the pulse wave had a tendency to smear the pulse wave feature points *s* and *dia*, and (ii) the breathing modulation of the pulse wave amplitude created some spreading on the pulse wave shape. In some applications, the normalisation of $t_{{\rm dia}}$ and CT with respect to the pulse wave width (i.e. IBI-PPG) sometimes provide better discrimination of a stress state and sometime not [28, 29].

### 3.2 Stress influences on ANS

It is well known that stress affects the ANS by mostly inhibiting the parasympathetic system and sometimes increasing or maintaining the sympathetic activity [20]. This results in changes in several measurable parameters such as HR and the RMSSD. Fig. 6 (upper panel) shows both parameters together with the VAS (%) results. Firstly, VAS increased significantly in the *Stroop 1* ($p = 10^{ - 6}$) and *Stroop 2* ($p = 0.7 \cdot 10^{ - 6}$) phases, and decreased in *relaxation* (not significant) from *baseline*. The perception of relaxation was felt strongly as shown by a large drop from *Stroop 1* to *relaxation* ($p = 0.0001$). No *VAS* were recorded during *recovery*. Secondly, *HR* increased significantly in *Stroop 1* ($p = 0.02$) and nearly significantly in *Stroop 2* ($p = 0.07$) with respect to *baseline*, and showed a greater variance in *Stroop 2* and *recovery* than in *Stroop 1*. Thirdly, RMSSD did not drop significantly in *Stroop 1* but did in *Stroop 2* ($p = 0.02$) with respect to *baseline*. It also increased its average from *baseline* (not significant) and variance during *relaxation*. Lastly, HR and RMSSD did not change significantly from *Stroop 2* to *recovery*.

**Fig. 7:**
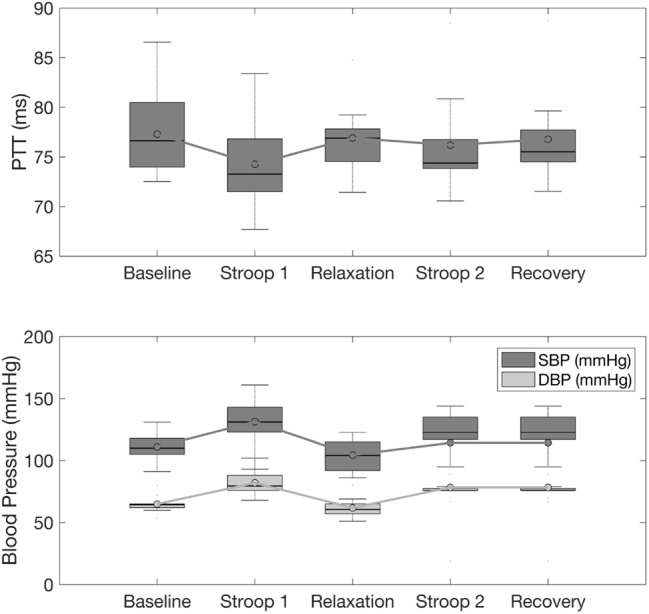
Influences of stress on (top) PTT, (bottom) SBP, and DBP

Stress also affected BP (Fig. 7, lower panel). Systolic BP (SBP) and diastolic BP (DBP) increased from *baseline* to *Stroop 1* ($p = 0.015$ and $p = 0.0004$, respectively) and decreased from *Stroop 1* to *relaxation* ($p = 0.0017$ and $p = 0.0009$, respectively). DBP also decreased from *baseline* to *relaxation* ($p = 0.0009$) and increased from *relaxation* to *Stroop 2* ($p = 0.0003$) and from *relaxation* to *recovery* ($p = 0.0003$). PTT average values changed across protocol phases but did not reach statistical significance, despite a considerable drop in the average values from *baseline* to *Stroop 1* and *Stroop 2*, and increase from *Stroop 1* to *relaxation* (Fig. 7, upper panel).

### 3.3 Pulse wave features

Fig. 6 (lower panel) shows the statistical behaviour of the averaged estimated pulse wave features $\widehat{{{\rm CT}}}$, $\hat t_{{\rm dia}}$, and $\widehat{{{\rm IPA}}}$ for each protocol phase across all subjects. We have superimposed the corresponding features calculated in our numerical study using the correspondence between in-vivo protocol phases and *in-silico* stress levels shown in Table 1 and the scaling factors shown in the figure caption.

We observed the following average behaviours. Firstly, the group average trends of $\hat t_{{\rm dia}}$ and $\widehat{{{\rm CT}}}$ from *baseline* to *relaxation* correlated well with the values obtained from our numerical study for stress levels *SSL*-1 to *SSL-*2 (Table 1). We obtained Pearson's correlation coefficients of 0.92 and 0.90, respectively, between the group average in-vivo and in-vitro parameters $t_{{\rm dia}}$ and CT. Secondly, $\hat t_{{\rm dia}}$ decreased from *baseline* to *Stroop 1* ($p = 0.01$) and increased from *Stroop 1* to *relaxation* ($p = 0.005$) and *Stroop 1* to *recovery* ($p = 0.003$); the drop from *relaxation* to *Stroop 2* was non-significant. Similarly, $\widehat{{{\rm CT}}}$ decreased from *baseline* to *Stroop 1* (near significance $p = 0.07$) and increased from *Stroop 1* to *relaxation* ($p = 0.016$) and *Stroop 1* to *Stroop 2* ($p = 0.04$). Thirdly, $\widehat{{{\rm IPA}}}$ showed a larger variability compared to $\hat t_{{\rm dia}}$ and $\widehat{{{\rm CT}}}$.

## 4 Discussion

The study of stress is challenging due to the lack of a gold standard assessment method, the difficulty in establishing a baseline condition, and the multi-factorial nature of the phenomenon which is affected by several confounding factors. We have focused on the cardiovascular impact of stress and shown that pulse waveform analysis of the PPG signal can be of complimentary use to HR and heart rate variability analysis. While the ANS and breathing responses to stress have been largely studied in the last two decades, the effects of stress on the pulse wave shape have only been recently studied.

Our first observation is that the percentage of high-quality pulse waves depends on the state of stress. Our criterion for qualifying pulse waves as high quality was the visual discrimination of the dicrotic notch, aided by visual inspection of the first and second derivatives. The fact that the highest percentage of high-quality signal was found in the relaxation phase indicates that in this phase the pulse wave is less distorted than in the other phases and in particular during the stress phases.

Our results show the potential of using PPG sensors to develop a technique for PPG-based monitoring of stress, in agreement with previous studies [13–15]. As shown in this study, PPG pulse wave features show lesser variance with stress levels than HR or heart rate variability, since pulse wave analysis is often affected by waveform distortion. Alternative methods for pulse wave analysis may solve the problem such as the harmonic analysis of short PPG segments, as shown by our recent study [30].

The miniaturisation and reduced costs of PPG sensors make this approach very appealing due to its versatility to measure and quantify the ANS state simultaneously with cardiac and vascular functions. Our results suggest that both ANS and pulse wave parameters can be used to assess stress and that both the average and variance of those parameters can be useful, which might indicate the potential use of higher-order statistics of ANS and pulse wave parameters for stress quantification. We hereafter discuss separately our findings on the impact of stress on indices associated with the ANS and the pulse wave.

### 4.1 ANS, PTT and BP

As expected, stress influenced the HR, parasympathetic activity (as measured using RMSSD), and BP with significant increase/decrease during stress/relaxation. The DBP showed a marked response with small variance across subjects as opposed to the SBP which had a larger variance across the entire protocol. We would thus recommend the use of the diastolic pressure as a feature for stress monitoring.

SBP has been shown to be linked to changes in PTT [31], but we did not find a statistically significant trend for PTT during the protocol. However, PTT and SBP showed similar variance across subjects potentially confirming their relationship.

Both HR and RMSSD were found to be markers of stress and relaxation, since their averages and variances changed significantly in both situations. We observed a physiological adaptation process throughout the study protocol: the effect of *Stroop 2* on the parameters PTT, SBP and DBP was less pronounced compared to corresponding effects of *Stroop 1* (Figs. 6 and 7).

### 4.2 Pulse wave analysis and validation of our *in-silico* study

Our previous computational study [18] on the effects of cardiovascular-activated stress on PPG-wave features identified three parameters that changed significantly with stress and relaxation: (i) $t_{{\rm dia}}$ (the time from dicrotic notch to end diastole); (ii) IPA (the ratio of diastolic to systolic area), and (iii) CT (the time from pulse onset to systolic peak). We have confirmed these predictions in this in-vivo study. Recent authors defined an index called *stress induced vascular response index* similar to IPA and was used as a measure of cognitive load [14, 15].

Changes in the *in-silico* parameters $t_{{\rm dia}}$ and *CT* were highly correlated with their in-vivo counterparts. The diastolic time ($t_{dia}$) decreased during stress and showed the least variance compared to all the other pulse wave parameters considered in this study. $t_{{\rm dia}}$ and CT decreased during stress showing that stress increases arterial stiffness as it impacts on reflected wave timing. IPA showed large variance across the protocol and subjects which can correlate with our *in-silico* results where a lowering trend of this parameter was shown with an irregular behaviour at different stress levels [18]. In our *in-silico* study we assessed the cardiovascular determinants of CT and $t_{{\rm dia}}$ [18]. We found that they are influenced not only by HR, but other cardiovascular properties too. This suggests that changes in these PPG features with stress are the result of multiple cardiac and vascular changes, rather than changes in HR alone.

Our results are also in agreement with [31] where CT and the pulse wave width (time from the foot of the wave to the dicrotic notch and, hence, directly related to $t_{{\rm dia}}$) were found relevant for stress assessment. The physiological adaptation process discussed in the previous section for the parameters PTT, SBP and DBP was also seen for $\widehat{{{\rm CT}}}$ (Fig. 6).

Despite the use of averaging of multiple pulse waves to reduce inter-beat variability, we sometimes found difficulties to find the dicrotic notch location in some subjects and some protocol phases. This problem is well known in pulse wave analysis which adds to the variability of the statistical estimates especially for ageing subjects or when the PPG is recorded in low blood perfused regions. The use of robust spectral or time-frequency/scale signal processing techniques may overcome this issue.

### 4.3 Multi-parameter and multi-scale approach to stress assessment

This study has shown that the pulse wave shape contains potent information to assess cardiovascular related mental stress which could be taken into account in wholistic approaches to this problem, in which this information would be merged with indices associated with the ANS (including breathing rate, HR, heart rate variability). Spectral and non-linear analysis of PPG signals has been shown to contain information relevant to health, making them potentially useful techniques for stress quantification [32, 33].

It is well known that stress influences many different organs and their functions, with cardiovascular and respiratory functions being two of the most important and vitals ones. Stress affects the breathing pattern (including rate and pulse wave features) [31]. Non-invasive techniques such as those based on solely or any combination of the electrocardiogram, PPG waves, and chest belt plethysmograms have been developed to measure and quantify the breathing patterns [34]. In this work, we have concentrated on the cardiovascular function and identified pulse wave features that could be integrated with respiratory and ANS indices for the development of robust stress assessment techniques. We have also observed the effect of breathing on the PPG pulse waveform. There are two main reasons for the influence of breathing on the pulse wave pattern: (i) breathing chest movements are propagated to the arm where the sensor is placed and (ii) breathing modifies the BP which in turn changes the HR and activates the peripheral sympathetic system stiffening the arteries, thus changing the various sub-waves timing composing the blood pulse wave. As these factors are important both from a signal processing and physiological point of views, we will consider them in a separate publication.

Stress is a multi-timescale phenomenon which impacts people's physiology and state at different time horizons, from minutes to weeks and even years [35]. It is therefore of great importance to take the next steps in understanding the long-term behaviour and interrelationships of ANS and pulse waveform indices in daily life on a larger database of subjects.

## 5 Conclusion

We have identified two pulse wave parameters that can be calculated from the PPG wave shape and which change significantly with mental stress and relaxation: the time from dicrotic notch to end diastole and the time delay from the foot of the pulse wave to the peak systole. Both showed statistical significance, showing that PPG wave analysis can be an additional tool for mental stress assessment.

PPG wave analysis has proved to be a valuable method to assess mental stress with commercially available sensors in the green part of the light-emitting spectrum. The placement of the sensor is of utmost importance for the sake of reproducibility, generalisation, and stability of the results. We found the arm location to be suitable. Resting condition without movement is also important to guarantee the best quality of the PPG signal. Pulse wave analysis has been shown to be of great power and complimentary to the HR, heart rate variability, PTT, BP, and psychological assessments for stress assessment.

Stress quantification is very challenging in the clinic and in daily lives due to many confounding factors. Nevertheless, our results show the potential of using high-quality PPG sensors to develop a robust technique for PPG-based monitoring of mental stress, especially for clinical use in patients suffering from cardiovascular stress. The miniaturisation and reduced costs of PPG sensors make this approach very appealing due to its versatility to measure and quantify simultaneously ANS state as well as cardiac and vascular function.

## 6 Funding and declaration of interests

This work was supported by a project grant from the British Heart Foundation (PG/15/104/31913) and the Wellcome/EPSRC Centre for Medical Engineering at King's College London (WT 203148/Z/16/Z). Polar Electro Oy partly financed this study. The views expressed are those of the authors and not necessarily those of the Wellcome Trust or EPSRC. P. Celka was an employee of Polar Electro Oy at the time of the study. The other authors report no conflicts.
